# Ligature-induced periodontitis induces systemic inflammation but does not alter acute outcome after stroke in mice

**DOI:** 10.1177/1747493019834191

**Published:** 2019-02-22

**Authors:** Conor O'Boyle, Michael J Haley, Eloise Lemarchand, Craig J Smith, Stuart M Allan, Joanne E Konkel, Catherine B Lawrence

**Affiliations:** 1 Faculty of Biology, Medicine and Health, Manchester Academic Health Science Centre, The University of Manchester, Manchester, UK; 2 Greater Manchester Comprehensive Stroke Centre, Manchester Academic Health Science Centre, Salford Royal NHS Foundation Trust, Salford, UK; 3 Manchester Collaborative Centre for Inflammation Research (MCCIR), Core Technology Facility, The University of Manchester, Manchester, UK

**Keywords:** Periodontitis, ischemic stroke, inflammation, oral disease, co-morbidities

## Abstract

**Background:**

Stroke is a major cause of disability and mortality. Poorer outcome after stroke is associated with concomitant inflammatory and infectious disease. Periodontitis is a chronic inflammatory disease of the dental supporting structures and is a prominent risk factor for many systemic disorders, including cardiovascular disease and stroke. While epidemiological studies suggest that periodontitis increases the likelihood of stroke, its impact on stroke severity is poorly understood. Here, we sought to determine the contribution of periodontitis to acute stroke pathology.

**Methods:**

We characterized a murine ligature model of periodontitis for inflammatory responses that could potentially impact stroke outcome. We applied this model and then subjected mice to either transient or permanent middle cerebral artery occlusion. We also enhanced the periodontitis model with repeated intravenous administration of a periodontal-specific lipopolysaccharide to better mimic the clinical condition.

**Results:**

Ligature-induced periodontitis caused bone loss, bacterial growth, and increased local inflammatory cell trafficking. Systemically, periodontitis increased circulating levels of pro-inflammatory cytokines, and primed bone marrow monocytes to produce elevated tumour necrosis factor-alpha (TNFα). Despite these changes, periodontitis alone or in tandem with repeated lipopolysaccharide challenge did not alter infarct volume, blood–brain barrier breakdown, or systemic inflammation after experimental stroke.

**Conclusions:**

Our data show that despite elevated systemic inflammation in periodontitis, oral inflammatory disease does not impact acute stroke pathology in terms of severity, determined primarily by infarct volume. This indicates that, at least in this experimental paradigm, periodontitis alone does not alter acute outcome after cerebral ischemia.

## Introduction

Stroke is a considerable cause of mortality and the leading cause of neurological disability, with post-stroke sequelae negatively impacting long-term mental and physical health.^1^ Despite a high prevalence and growing incidence, current stroke treatments are limited to reperfusion strategies that, though effective in some patients, are not widely applicable. Lack of alternative stroke treatments is due to a number of reasons, one of which is an under-appreciation of the role that co-morbidities play in stroke etiology and prognosis.^2^ It is clear that pre-existing conditions, often with inflammatory pathogeneses, are major determinants of stroke-induced damage in both humans and rodent models. Indeed, obesity,^3–5^ infection,^6,7^ atherosclerosis,^8^ and old age^9,10^ can all negatively affect ischemic brain damage and overall recovery in animal models. Although there is an emerging focus on stroke co-morbidities, the common oral disease periodontitis (PD) is under-studied in this respect, despite being frequently associated with increased risk of ischemic stroke in humans.^11–20^

PD is a chronic inflammatory condition that affects the supporting structures of the dentition, and is considered one of the most prevalent inflammatory diseases in humans.^21,22^ PD involves formation of a deep ulcerated pocket that harbors proliferating anaerobic bacteria, which leads to chronic inflammation and destruction of the supporting bone. Up to 50% of the global population is affected by some form of periodontal disease and as much as 10% affected by severe PD, which can adversely affect systemic health.^23,24^ Indeed, PD is emerging as a possible risk factor for many systemic diseases,^25^ including diabetes,^26^ rheumatoid arthritis,^27^ Alzheimer's disease,^28–31^ and cardiovascular disease.^32–34^ Pre-clinical studies have directly implicated PD in the development and progression of atherosclerosis,^35–39^ which is intimately linked to increased incidence of stroke.

There are a number of proposed mechanisms that explain how pathophysiological events in the oral cavity affect the cardiac vasculature and distant tissue sites. During PD, ulceration of the epithelium and lysis of deeper periodontal tissues facilitates greater translocation of oral bacteria or their products (e.g. lipopolysaccharide (LPS)) into the bloodstream which can induce systemic inflammation or endotoxemia.^40,41^ Similarly, pro-inflammatory mediators from the periodontium can elicit systemic effects if they “spill over” into the bloodstream.^11,21^ Chronic PD is known to result in increased and sustained levels of pro-inflammatory mediators in the systemic circulation.^42^ Further, the healthy brain with an intact blood–brain barrier (BBB) is susceptible to systemic inflammatory challenge.^43^ A compromised BBB, as occurs in stroke, may facilitate entry of pro-inflammatory molecules and/or bacterial products and propagate damage within the brain. LPS from the prominent periopathogen *Porphyromonas gingivalis* (*P. gingivalis*) has been found in the demented brain.^30^ Furthermore, periodontal pathogens themselves are highly invasive; *P. gingivalis* can compromise and cross the BBB into the brain,^44^ and others (*Treponema* spp.) have been speculated to enter the brain directly via peripheral trigeminal nerves.^31^ Taken together, this indicates that chronic periodontal injury may modulate events in the brain post-stroke and affect outcome.

PD and stroke share similar risk factors, such as age, smoking, hypertension, and obesity,^45^ and both conditions share inflammatory pathways. Further, a systemic pro-inflammatory profile pre- and post-ischemia is an important determinant of poorer outcome.^7,46–48^ Although epidemiological studies suggest that PD is associated with increased stroke risk, there is a lack of pre-clinical research on whether PD worsens outcome after stroke.

### Aims

The aim of the present study was therefore to determine the contribution of the prevalent oral disease PD to ischemic brain damage. We employed a clinically relevant model of PD in mice and determined its effect on peripheral and central inflammation and acute stroke outcome by using two different models of cerebral ischemia.

## Materials and methods

### Animals

Male 8–12-week-old C57BL/6 mice (Envigo, UK) were used for all studies. Animals were housed in individually ventilated cages (temperature 21 ± 2℃; humidity 55% ± 5%; 12-h light/12-h dark cycle) and given access to food and water *ad libitum* under specific pathogen-free conditions. Animals were allocated treatments and surgical fates in a randomized order across cages. The order of surgeries was also randomized. All scientific procedures were performed in accordance with the Animals (Scientific Procedures) Act 1986 under relevant UK Home Office licences and approved by the local Animal Welfare and Ethical Review Board (University of Manchester, UK).

### Ligature-induced PD and LPS challenge

Procedures described here are adapted from Abe and Hajishengallis.^49^ Briefly, mice were anaesthetized via the intraperitoneal route with a mix of Narketan (50 mg/kg; ketamine) and Domitor (650 µg/kg; medetomidine) (both Vétoquinol, UK). Silk ligatures (5–0, Fine Science Tools, Germany) were tied around both second maxillary molars. Mice were revived with subcutaneous administration of Antisedan (1 mg/kg; atipamezole) (Vétoquinol) and monitored until recovery. “Control” animals were anesthetized only and not subjected to creation of interdental spaces or ligature placement (as to do so would cause damage that is associated with PD). Bone loss in this model occurs at 10 days post-ligature placement. Ligatures in place longer would risk teeth falling out which is a clear exclusion criterion. There was no mortality during or post-surgery. Where applicable, LPS from *P. gingivalis* (Pg-LPS) (1 mg/kg; InvivoGen, France) was administered intravenously at days 1, 3, 5, 7, 9 post-ligature placement.

### Bone loss measurements

For bone loss, mice were euthanized via CO_2._ Heads were then removed and defleshed, and maxillae were isolated and stained with 1% methylene blue. Images of the palatal side of the maxillae were taken using a Leica Stereo Fluorescence M205 FA (Leica Microsystems, UK). Quantification of bone loss was then calculated across six different molar sites by measuring the distance from cemento-enamel junction (CEJ) and alveolar bone crest (ABC) using Image J (NIH, USA).

### Bacterial colony-forming units

Swabs of the oral cavity were taken at time of sacrifice and placed into sterile phosphate buffered saline (PBS). Dilutions were plated on trypticase soy agar (Oxoid, UK) and incubated at 37℃ for 24 h to detect non-selective growth of aerobic bacterial species. To detect anaerobic growth, oral swab suspensions were plated on Wilkins-Chalgrens agar (Oxoid, UK) and placed in a hypoxic chamber at 37℃ for 72 h. Colony-forming units (CFUs) were determined by manually counting bacterial colonies.

### Filament middle cerebral artery occlusion

Focal cerebral ischemia was induced via transient occlusion of the middle cerebral artery by filament (fMCAo) as described previously.^50^ Briefly, under isoflurane anesthesia (4% induction, 1.5–2% maintenance, in a 70:30 mix of N_2_O:O_2_), the carotid arteries were exposed and a 6/0 silicon-coated nylon filament (Doccol, USA) was introduced into the internal carotid artery and advanced to occlude the middle cerebral artery (MCA) at its origin. A laser Doppler probe (Moor Instruments, UK) was used to monitor cerebral blood flow (CBF). Successful occlusion was confirmed by reduction in CBF of approximately 80% or greater. The filament was withdrawn after 20 min to allow reperfusion and the wound sutured. Mice were normothermic prior to surgery and during surgery core body temperature was maintained at 37 ± 0.5℃. Post-surgery, buprenorphine (0.05 mg/kg) for analgesia and 0.5 ml saline were administered subcutaneously and mice were placed in a warm cabinet (27–28℃) to recover. Animals were excluded from analyses if there was absence of stroke (*n* = 1) or mortality during surgery (*n* = 1).

### Distal middle cerebral artery occlusion

Permanent occlusion of the distal MCA (dMCAo) was induced as described previously.^51^ Briefly, mice were anaesthetized with isoflurane (5% induction, 2–2.5% maintenance, in a 70:30 mix of N_2_O:O_2_) and mounted on a stereotactic frame. The temporal muscle was detached from the skull and a small cranial window drilled directly above the MCA. A triangle of filter paper soaked in freshly prepared 30% ferric chloride (FeCl_3_) was applied to the distal MCA at a bifurcation above the zygomatic arch and left in place for 5 min. A platelet-rich thrombus formed *in situ* and was allowed to develop for 45 min until it fully occluded the vessel. A laser Doppler probe (Moor Instruments) was used to monitor CBF. Successful occlusion was confirmed by a stable CBF reduction of approximately 70% or greater. During surgery, core body temperature was maintained at 37 ± 0.5℃. Buprenorphine (0.05 mg/kg) for analgesia and 0.3 ml saline were administered subcutaneously post-surgery. Animals were excluded from analyses if there was absence of stroke (*n* = 4).

### Infarct volume

Under terminal isoflurane anesthesia, animals were perfused with PBS and subsequently perfuse-fixed with 4% paraformaldehyde (PFA). Brains were excised, post-fixed in 4% PFA, and then immersed in 30% sucrose for cryoprotection before freezing at −80℃. Coronal brain slices (30 µm) were sectioned on a freezing sledge microtome and mounted on gelatin-coated slides, and then stained with 1% cresyl violet. For dMCAo, infarcted areas, denoted by pale staining, were calculated by total area × thickness of section. For fMCAo, infarcted areas were identified and were drawn onto corresponding brain maps at eight pre-determined coronal levels (2.22, 1.54, 0.86, 0.14, −0.58, −1.22, −1.94, −2.7 mm according to Bregma).^52^ Total infarct volume was determined by calculating area under the curve using GraphPad Prism v7.

### Immunohistochemistry

Coronal brain sections (30 µm) were used for all subsequent immunohistochemistry procedures. Endogenous peroxidase activity was quenched with 1% H_2_O_2_, followed by 5% goat serum to prevent non-specific antibody binding. Anti-neutrophil serum SJC-4 (1:10,000) (kindly provided by Professor D. Anthony, University of Oxford, UK) was applied overnight at 4℃, followed by a biotinylated goat anti-rabbit secondary antibody (1:500; Vector Laboratories, UK). Amplification of signal was achieved by a Vectastain ABC-HRP kit (Vector Laboratories) and visualization of positive staining was achieved with 3,3′-diaminobenzidine (DAB) (Sigma-Aldrich, UK). For IgG staining, immunohistochemistry was performed as above but omitting the primary antibody step. Images were scanned on a Pannoramic 250 Flash III slide scanner (3DHISTECH, Hungary) and SJC-4-positive neutrophils were quantified at 5 × magnification in the entire area of striatum and/or cortex with Image J/Fiji. Counts were averaged over three coronal levels (0.86, 0.14, 0.58 mm according to Bregma)^52^ and expressed as neutrophils/section. IgG staining intensity in the infarcted hemisphere was calculated as the change in pixel contrast from the contralateral side and averaged across three coronal levels (0.14, −0.58, −1.94 mm according to Bregma).^52^

### Plasma cytokine determination

Blood was centrifuged at 1500 × *g* for 10 min to isolate plasma. Multiplex analysis of cytokine production was determined using LEGENDplex (Biolegend), according to the manufacturer's instructions. Samples were acquired on a FACSVerse flow cytometer (BD Biosciences) and analyzed using the LEGENDplex v8 software. In a minority of samples that did not yield detectable cytokine levels, these samples were assigned the lowest limit of detection.

### Preparation of single cell suspensions

Blood, bone marrow, spleen, and sub-mandibular lymph nodes (SMLNs) were collected in RPMI 1640 medium (Sigma) supplemented with HEPES (1M; Sigma), Penicillin–Streptomycin (Sigma), and 3% fetal bovine serum (FBS) (Life Technologies). Spleens and SMLNs were mashed through a 70 µm filter (Fisherbrand, UK) to yield a single cell suspension, followed by red blood cell lysis (Lonza, UK). Blood was subjected to two red blood cell lyses. Bones were opened at the knee joint and centrifuged to collect the marrow, which subsequently underwent red blood cell lysis.

### Flow cytometry

Single cell suspensions were stained with Fc receptor block (TruStain fcX, Biolegend) along with the appropriate anti-mouse antibodies: CD45 (AF700; clone 30-F11, Biolegend), TNFα (AF700; clone MP6-XT22, Biolegend), CD11b (BV605 or BV650; clone M1/70, Biolegend), Ly6C (BV711; clone HK1.4; Biolegend), Ly6G (APC-Cy7; clone 1A8), CD115 (APC; clone AFS98, Biolegend) or PE-Cy7; AFS98, eBioscience), CD68 (PE; clone FA-11, Biolegend). A lineage gate was used to exclude other cell populations and consisted of the following biotinylated antibodies: CD3 (clone 145-2C11, Biolegend), CD19 (clone 6D5, Biolegend), NK1.1 (clone PK136, Biolegend), Ter119 (clone TER-119, Biolegend), and SiglecF (PE-CF594; E50-2440, BD Biosciences). A fixable stain (LIVE/DEAD Blue, Life Technologies) was used to exclude dead cells. For intracellular staining, cells were first stained for surface markers, fixed in 2% PFA, then permeabilized in 0.5% Saponin (Sigma), and stained for intracellular markers. Fluorescence-minus-one controls were used to validate results. Samples were acquired on a Fortessa flow cytometer (BD Biosciences) and analyzed using FlowJo software v10 (FlowJo LLC, USA).

### Ex vivo re-stimulation for cytokine induction

Bone marrow cells were stimulated with or without LPS (10 ng; O111:B4 Ultrapure, InvivoGen) in 10% FBS-containing media in the presence of Brefeldin A (5 µg/ml; BD Biosciences) for 2.5 h at 37℃ and then stained for flow cytometry.

### Data and statistical analyses

All statistical analyses were performed using GraphPad Prism v7 using the appropriate test (stated in the figure legends). Data were transformed where appropriate. All data are presented as SEM. *p* < 0.05 was considered statistically significant. Sample sizes were determined by power calculation (α = 0.05, β = 0.2) of our previous data. All ex vivo quantifications were performed in a blinded manner. All reporting of animal experiments complied with the ARRIVE guidelines (Animal Research: Reporting *In Vivo* Experiments).

## Results

### Ligature-induced PD causes robust pathology in the oral cavity

**Figure 1.**
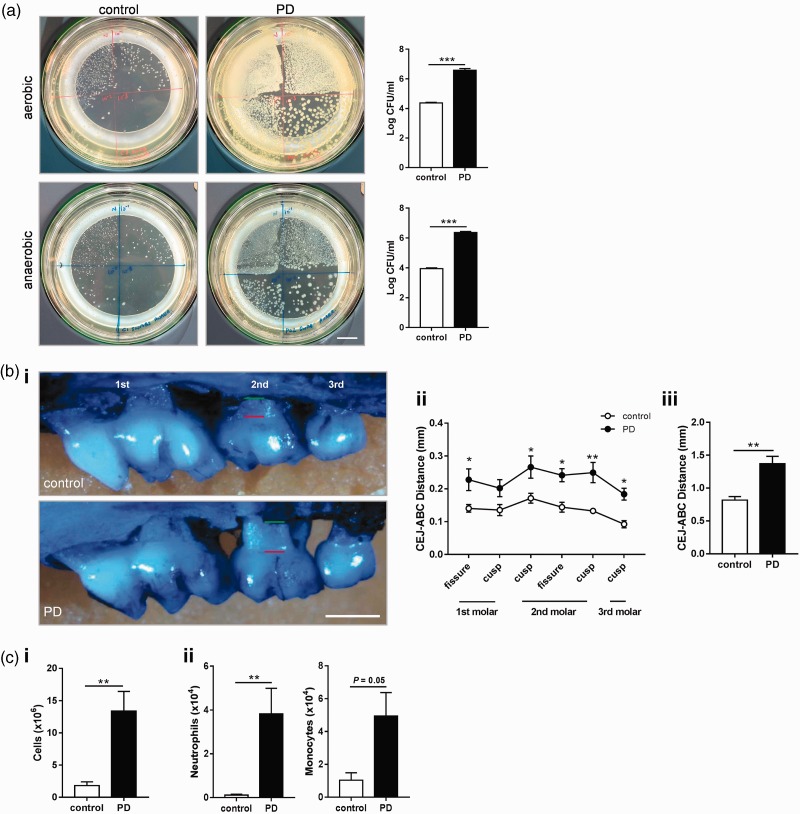
Ligature-induced periodontitis causes robust pathology in the oral cavity. To induce periodontitis (PD), mice were subjected to ligatures placed bilaterally around the second maxillary molars for 10 days or control surgery. (a) Oral swabs from periodontitis (PD) and control mice taken at day 10 were plated under aerobic conditions for 24 h or anaerobic conditions for 72 h. Scale bar, 1 cm. (b) i. Representative images of maxillae showing bone loss at day 10 post-ligature placement. *Green line*: alveolar bone crest (ABC). *Red line*: cemento-enamel junction (CEJ). Scale bar, 0.5 mm. (b) ii. Individual sites of bone loss (b) iii. Total bone loss was measured by sum of the CEJ-ABC distances across the six molar sites. (c) i. Sub-mandibular lymph nodes were excised and overall cellularity determined. (c) ii. Total numbers of neutrophils and monocytes were determined by flow cytometry (for gating strategy, see Figure 2(b)). (a–c): **p < 0.01, ***p < 0.001, by unpaired Student's *t*-test. (b) ii. *p < 0.05, **p < 0.01 by two-way ANOVA with Sidak's *post hoc* test). *n* = 4–5 per group. N.B.: one aerobic PD plate was overgrown and not able to be counted and thus omitted.

Placement of ligatures around the second maxillary molars provides a reservoir for bacterial growth and also prevents habitual cleaning, culminating in profound bone loss within an acute timeframe. Indeed, within 10 days of ligature placement, there was a significant outgrowth of aerobic and anaerobic bacteria in the PD group (Figure 1(a)). Robust bone loss was also observed in the PD mice predominantly at sites around the second molar (Figure 1(b)). In the draining lymph nodes for the oral cavity, the sub-mandibular lymph nodes, there was a 6-fold increase in overall cellularity, and a significant influx of neutrophils and monocytes (Figure 1(c)). Combined, ligature placement was an effective model of PD, activating bacterial and host pathways that drive predictable periodontal bone loss in an acute timeframe.

### Ligature-induced PD causes systemic inflammatory changes

**Figure 2.**
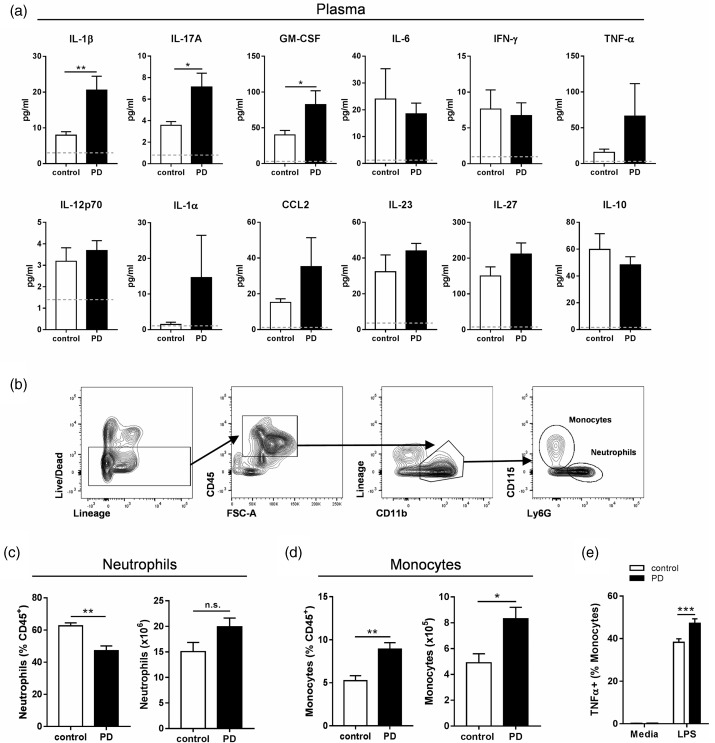
Ligature-induced periodontitis causes systemic inflammatory changes. To induce periodontitis (PD), mice were subjected to ligatures placed bilaterally around the second maxillary molars for 10 days or control surgery. (a) Plasma cytokine levels were determined by LEGENDplex assay. Grey dotted lines indicate the limit of detection for this assay. (b) General flow cytometry gating strategy for bone marrow monocytes^#^ (Live, CD45^+^, Lineage^−^, CD11b^+^, CD115^+^) and neutrophils (Live, CD45^+^, Lineage^−^, CD11b^+^, Ly6G^+^). (c,d) Frequency and numbers of neutrophils and monocytes in the bone marrow. (e) Ex vivo TNF-α production by bone marrow monocytes after 2.5 h stimulation with 10 ng LPS. ((a)–(d): *p < 0.05, **p < 0.01 by unpaired Student's *t*-test. (e) ***p < 0.001, by two-way ANOVA with Sidak's post hoc test). *n* = 4 per group, except (a) *n* = 9–10 per group. ^#^For cytokine production, monocytes were gated CD68^+^ instead of CD115^+^.

Clinically, PD is proposed to impact systemic health.^53^ However, there is limited evidence that ligature models in rodents induce inflammation outside the oral cavity. Here we show that there are systemic inflammatory consequences at 10 days after PD induction. The pro-inflammatory cytokine interleukin-1beta (IL-1β) was significantly elevated in the plasma of animals with experimental PD (Figure 2(a)). There was also a significant increase in both plasma IL-17A and granulocyte-macrophage colony-stimulating factor (GM-CSF) levels in the PD group, cytokines that mediate granulocyte and monocyte responses. Despite increases in GM-CSF and IL-17A, there was no difference in the overall number of bone marrow neutrophils, indicating that production of these cells was unaltered (Figure 2(c)). However, there was an increase in monocyte production in the bone marrow of PD animals (Figure 2(d)), and these monocytes were primed to produce more TNF-α (Figure 2(e)). Together, these data show that ligature-induced PD mediates specific inflammatory effects at peripheral sites distal from the oral cavity.

### Ligature-induced PD does not alter ischemic brain damage after filament MCAo

**Figure 3.**
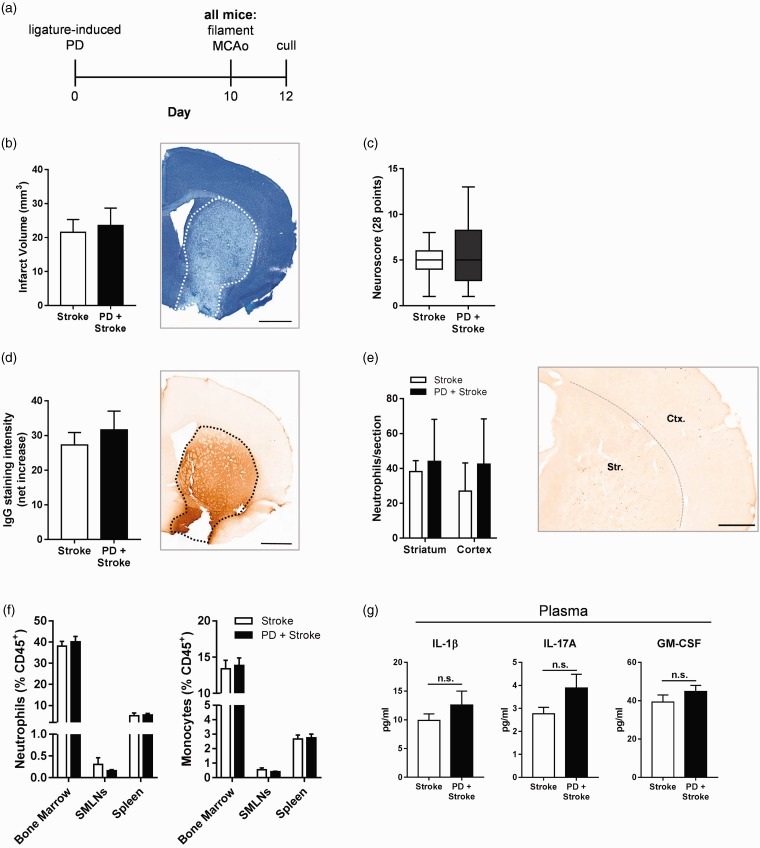
Ligature-induced periodontitis does not alter ischemic brain damage after filament middle cerebral artery occlusion. Mice were subjected to either ligature-induced periodontitis (PD) or control surgery for 10 days upon which they were given a 20-min filament middle cerebral artery occlusion (fMCAo) and sacrificed 48 h later. (a) Experimental setup. (b) Total infarct volume was determined via cresyl violet staining. Representative image of section with dotted line representing area of infarct. Scale bar, 1 mm. (c) Neurological impairment at 48 h was evaluated by 28-point neurological score. (d) Blood–brain barrier breakdown assessed by IgG immunohistochemistry. Representative image of section with dotted line representing area of infarct. Scale bar, 1 mm. (e) SJC-4 ^+^ neutrophils were counted in the striatum (Str.) and cortex (Ctx.). Scale bar, 500 µm. (f) Peripheral monocytes and neutrophil frequencies were assessed by flow cytometry. (g) LEGENDplex analysis of plasma cytokine levels. *n* = 10–11 per group. SMLNs: sub-mandibular lymph nodes.

In order to evaluate the impact of PD on stroke outcome, mice were given ligature-induced PD for 10 days followed by fMCAo (Figure 3(a)). As expected, fMCAo resulted in infarction of the striatum, and extended to the cerebral cortex in some animals. However, there was no difference in infarct volume between mice with or without PD assessed at 48 h (Figure 3(b)). In addition, there was also no difference in sensory-motor functional assessment between stroked mice with or without PD (Figure 3(c)). BBB breakdown occurred after stroke yet IgG staining intensity in the ipsilateral hemisphere was not significantly affected by PD (Figure 3(d)). Despite alterations in myeloid trafficking in the periphery in PD-only animals (Figures 1 and 2), neutrophil infiltration in the ipsilateral hemisphere post-stroke was similar between the groups (Figure 3(e)). In the peripheral compartment, PD did not significantly modulate myeloid trafficking compared to the levels observed in the stroke-only group (Figure 3(f)), nor were plasma concentrations of IL-1β, IL-17A, and GM-CSF altered (Figure 3(g)). Further, the levels of B cells and T cells in the peripheral sites shown were also unchanged (data not shown). Taken together, these data show that PD does not modulate any of the assessed acute outcomes in a transient model of focal cerebral ischemia.

### Ligature-induced PD does not alter ischemic brain damage after distal MCAo

**Figure 4.**
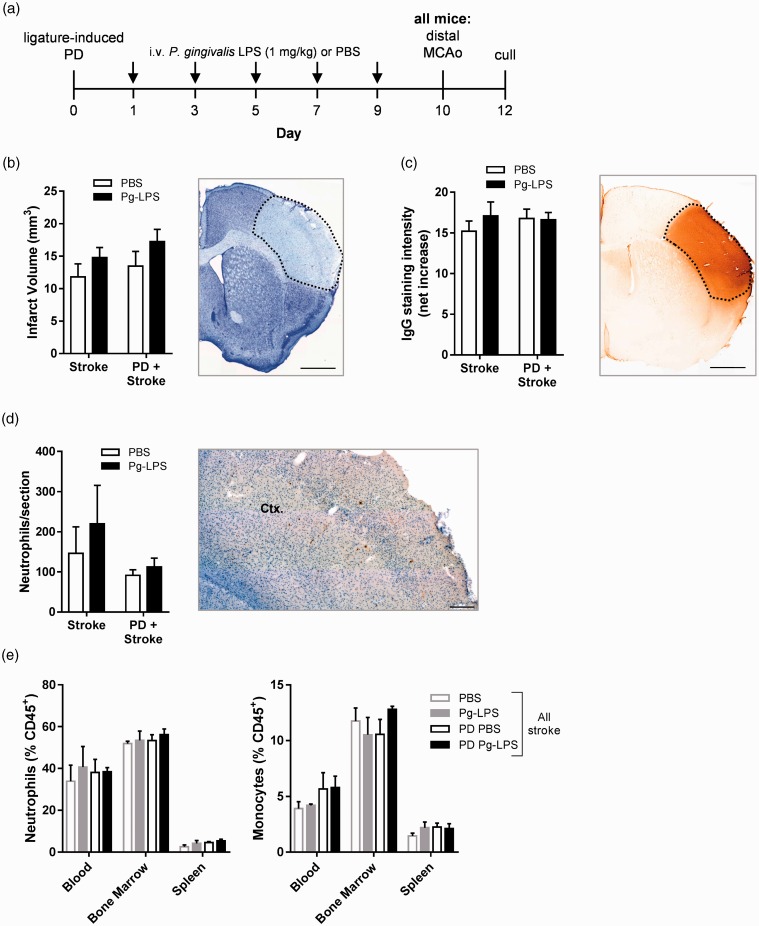
Ligature-induced periodontitis does not alter ischemic brain damage after distal middle cerebral artery occlusion. Mice were subjected to either ligature-induced periodontitis (PD) or control surgery in tandem with intravenously administered repeated low-dose (1 mg/kg) *Porphyromonas gingivalis* LPS (Pg-LPS) or PBS for 10 days, upon which they were given a distal middle cerebral artery occlusion (dMCAo) and sacrificed 48 h later. (a) Experimental setup and dosing strategy. (b) Total infarct volume was determined via cresyl violet staining. Representative image of section with dotted line representing area of infarct. Scale bar, 1 mm. (c) Blood–brain barrier breakdown assessed by IgG immunohistochemistry. Representative image of section with dotted line representing area of infarct. Scale bar, 1 mm. (d) SJC-4 ^+^ neutrophils were counted in the cortex (Ctx.). Scale bar, 200 µm. (e) Peripheral monocytes and neutrophil frequencies were assessed by flow cytometry. *n* = 5–9 per group, except (e) *n* = 3–5 per group.

As ligature-induced PD did not affect acute outcome in a transient model of focal cerebral ischemia, we employed a permanent model to determine if experimental PD could affect stroke pathology in a permanent model of stroke (dMCAo) and consolidate the previous finding. We also intravenously administered repeated doses of LPS from *P. gingivalis* (Pg-LPS) concomitantly with ligature-induced PD to better reflect the systemic inflammatory changes reported in PD patients.^54^ After dMCAo, however, PD had no effect on ischemic brain damage or on BBB breakdown (Figure 4(b) and (c)). Administration of Pg-LPS also had no effect on infarct volume or BBB permeability in either control or PD mice. Neutrophil infiltration into the ipsilateral cortex was also unaffected by Pg-LPS and/or PD (Figure 4(d)). Moreover, in the blood, bone marrow, and spleen, neutrophils and monocytes were not significantly altered by either Pg-LPS and/or PD following dMCAo (Figure 4(e)). B and T cell levels in these peripheral sites were also unchanged (data not shown). Together, these data show that experimental PD does not alter acute outcome after dMCAo.

## Discussion

PD has been implicated as a factor for increased incidence of stroke but whether PD can modify ischemic brain damage is not fully understood. The present study shows that ligature-induced PD induced robust bone loss in an acute timespan accompanied by local and systemic inflammation that is comparable to the clinical condition. However, in both permanent (dMCAo) and transient (fMCAo) models of focal cerebral ischemia, PD did not alter infarct volume, BBB breakdown, or acute inflammatory processes.

The double ligature model of PD is an attractive tool for experimental periodontal research.^49^ Bone loss is the hallmark feature of PD in humans. These changes are also accompanied by bacterial outgrowth and local inflammation. We found that ligature-induced PD successfully mimics these clinical characteristics; bone loss occurred in an acute timeframe, in agreement with other reports,^49,55,56^ and this was accompanied by profound bacterial expansion and changes in local inflammatory cell trafficking. This model is thus more favorable than other established models of PD, such as oral inoculation with *P. gingivalis*, which does not always yield robust bone loss and require antibiotic pre-treatment in order for *P. gingivalis* successfully colonize.^57–59^ A predictable timescale with human-relevant pathology makes ligature-induced PD a tractable model for studying the pathophysiology of PD.

PD is attributed to increased risk of inflammatory diseases but direct causal evidence is lacking. In-depth analyses of peripheral tissues and immune populations have not been given much focus in ligature-induced rodent models. Here, we report that ligature-induced PD caused a specific increase in plasma IL-1β, IL-17A, and GM-CSF levels. These cytokines have been implicated as key drivers of PD pathogenesis,^60^ but IL-17A and GM-CSF have not been previously reported in the circulation in animal models. These changes during PD have potentially important implications for stroke as systemic inflammation unequivocally worsens ischemic damage,^46,47^ and IL-1β has a well-documented central role in the acute phases post-stroke.^43,47,61^ IL-17A has also been implicated in perpetuating damage after stroke; trafficking of IL-17^+^ γδ T cells to the ischemic brain is associated with worse outcome.^62–64^ In contrast, GM-CSF is reportedly neuroprotective after stroke,^65^ indicating a potentially complex role for the IL-17A:GM-CSF axis in ischemic brain damage.

Many studies have also highlighted the contribution of neutrophil and monocyte trafficking to the ischemic brain; acute neutrophil infiltration is associated with increased brain damage, whereas monocyte recruitment can be detrimental or beneficial.^47,48,66,67^ We found that neutrophils and monocytes were mobilized to local lymph nodes during PD, and monocytes in the bone marrow increased in number and were primed to produce elevated TNF-α. This suggests that one way in which PD could mediate peripheral inflammation is by affecting monocyte phenotype as monocytes can be primed prior to bone marrow egress.^68^ This is in agreement with a study by Miyajima et al.,^69^ in which ligature-induced PD promoted monocyte/macrophage activation and adherence to aortic walls. Interestingly, the authors also found elevated TNF-α signalling components in the aortas of PD mice,^69^ but no change in serum TNF-α levels, which agrees with our finding that plasma TNF-α levels did not change in PD mice, despite a change in monocyte phenotype. This indicates that the peripheral effects of experimental PD are “low-grade” and only affect certain cell populations or certain tissue sites and do not induce multi-site “high-grade” pro-inflammatory responses.

Though high-grade systemic inflammation is deleterious in the context of stroke,^46–48^ there are contrasting reports on whether low-grade inflammation is harmful or protective after cerebral ischemia. Obesity in mice (which is associated with low-grade inflammation) increases ischemic brain damage, BBB breakdown, and also increases neutrophil infiltration into the ischemic territory.^3–5^ However, in rats, mild systemic inflammation induced by ligature-induced PD has been reported to have neuroprotective effects, supressing macrophage accumulation in the brain after stroke.^70^ The latter study conflicts with our data where we show PD did not alter ischemic brain damage. We did not find elevated levels of plasma IL-10 and interferon (IFN)-γ in PD animals, in contrast to Petcu et al.^70^ The use of a different species (rat), longer PD timeframe (15–21 days), and a different stroke model as well as longer reperfusion time (7 days) could account for the differing results. However, our study has the advantage of using two different stroke models and a clinically relevant LPS to consolidate our findings.

Transient MCAo by intraluminal filament is a well-characterized and well-established experimental murine model of stroke that mimics severe cerebral ischemia in humans.^71^ Importantly, ischemic damage and the inflammatory response can be modulated by the presence of co-morbidities, allowing dissection of the pathophysiological mechanisms after stroke.^3,6,7^ However, in the present study, PD did not drive ischemic damage above control levels; this is likely due to the fact that other studied co-morbidities involve major infection,^6,7^ or major metabolic shift (i.e. obesity),^3,72^ which may act as severe systemic stressors in the context of stroke in a manner that experimental PD does not. With this in mind, we sought to enhance the systemic inflammatory response associated with PD by administering the oral-specific Pg-LPS, as over 50% of PD patients have Pg-LPS in the bloodstream.^54^ Additionally, a permanent occlusion model (dMCAo) was employed, wherein the infarcts are smaller and confined to the cortex.^73^ However, neither ligature placement nor Pg-LPS affected ischemic brain damage in this model. Other *in vivo* studies have used lower efficacious Pg-LPS dosing regimes than that of the present study,^74^ which would indicate that biological efficacy of the Pg-LPS dose is not in question. However, it is plausible that tolerance to Pg-LPS, which can occur with repeated low doses,^75,76^ could explain the lack of significant change in stroke severity.

The acute timeframe of the PD model must be considered, as this may not lead to sufficiently chronic inflammatory changes to affect stroke pathophysiology. Certainly, human PD is a chronic condition associated with transient bacteremia and low-level inflammation that occurs over a long period.^40^ However, ligatures left in place for longer would risk tooth loss and thus a more suitable PD model may need to be developed that encapsulates local and systemic pathology in a chronic manner. Importantly, the mice used in this study were young and otherwise healthy, and most individuals with PD have other confounding factors (smoking, hypertension, old age, obesity) which can independently account for increased stroke risk.^2^ As experimental PD did not alter outcome in the present study, it indicates that PD alone is not sufficient to modulate the pathological changes associated with stroke. It remains to be determined, however, if PD increases the likelihood of stroke, if PD affects long-term recovery after stroke, and also, if in tandem with other co-morbidities PD worsens prognosis post-stroke. Such questions are difficult to address in experimental animal models, but given the high prevalence of PD and the growing incidence of stroke, they are important avenues that require future study.
